# Supplementary material for: Tensor image registration library: Deformable registration of stand‐alone histology images to whole‐brain post‐mortem MRI data
Source: Neuroimage. 2023 Jan;265:119792. doi: 10.1016/j.neuroimage.2022.119792 (PMC10933796; doi:10.1016/j.neuroimage.2022.119792)
Supplement: Supplementary file 1 [file mmc1.docx]

**Supplementary Material 1 –**

**Details of the ANTs registrations that were used in the Stage-1 accuracy comparison testing**

Comparisons were made with both the Mattes mutual information and the cross-correlation metrics, that were used in a previous study [25] to register histology sections. The registrations were carried out on the same 14 callosal and 14 hippocampal sections that were previously registered to their corresponding tissue blocks with the Stage 1 routine. For a fair comparison, all ANTs registrations were initialised using antsAffineInitializer with 30° increments over the whole circle. Various combinations of parameters were screened for each metric, starting from those that were recommended in the ANTs documentation. Empirically the following multi-resolution configurations were found to yield the best results with ANTs:

1. ANTs SyN Mattes: antsRegistration –dimensionality 2 –float 0 –output $outdir/ants.syn/moving_to_fixed –interpolation Linear –winsorize-imageintensities [0.005,0.995] –use-histogram-matching 1 -r $outdir/ants.syn/init.mat -m Mattes[$outdir/fixed.png, $outdir/moving.png,1, 20, Random, 0.2] -t affine[2.0] -c [1500 x 1500 x 1500 x 300 x 100 x 0, 1.e-7, 5] -s 5x4x3x2x1x0 -f 7x6x5x4x2x1 -m Mattes[$outdir/fixed.png, $outdir/moving.png, 1, 32] -t syn[0.25,3.0,1] -c [200 x 200 x 200 x 200 x 150 x 50, 0, 5] -s 5x4x3x2x1x0 -f 7x6x5x4x2x1
2. ANTs SyN CC: antsRegistration –dimensionality 2 –float 0 –output $outdir/ants.syn.cc/moving_to_fixed –interpolation Linear –winsorize-image-intensities [0.005,0.995] –use-histogram-matching 1 -r $outdir/ants.syn.cc/init.mat -m Mattes[$outdir/fixed.png, $outdir/moving.png, 1, 20, Random, 0.2] -t

As the TIRL Stage 1 routine uses binary masks for the registration, the masks were exported from the TIRL pipeline, and the ANTs registrations were repeated with the masks. The previously generated contours of the tissue block photographs were transformed to histology space with the antsApplyTransformsToPoints tool, and the MCDs were calculated to measure the accuracy of the registrations in each case:

antsApplyTransformsToPoints –dimensionality 2 –precision 1 –input blockpts − −outputd/transformed_block_contour.csv -t [$d/moving_to_fixed0GenericAffine.mat,1] -t $d/moving_to_fixed1InverseWarp.nii.gz
