# Supplementary material for: Tensor image registration library: Deformable registration of stand‐alone histology images to whole‐brain post‐mortem MRI data
Source: Neuroimage. 2023 Jan;265:119792. doi: 10.1016/j.neuroimage.2022.119792 (PMC10933796; doi:10.1016/j.neuroimage.2022.119792)
Supplement: Supplementary file 2 [file mmc2.docx]

**Supplementary Material 2 –**

**Example Stage-3 registration of a severely damaged coronal brain s using a manually defined binary mask for cost-function weighting.**


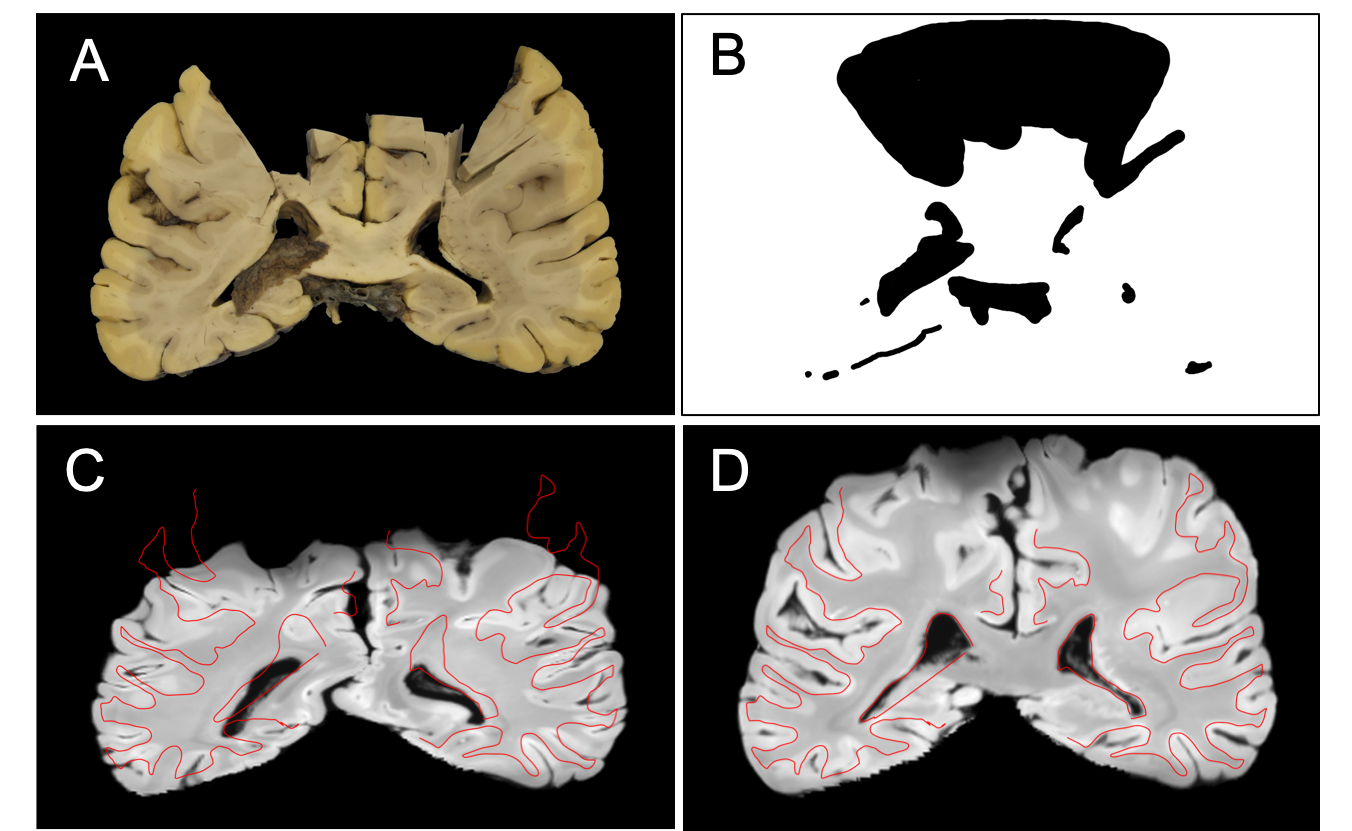


**Figure B.1. Result of slice-to-volume registration of a severely damaged coronal brain slab.** **(A)** Coronal brain slab photograph with bilateral hiatus in the sensorimotor regions. **(B)** A hand-drawn binary mask for cost-function weighting. **(C)** Registration result without using the target mask. The *red curve* is an overlay of the manually segmented grey-white matter contour of the brain slab photograph. **(D)** Registration result with the hand-drawn target mask. The accuracy of the corrected registration is qualitatively similar to that on non-damaged slices.
