# Supplementary material for: Tensor image registration library: Deformable registration of stand‐alone histology images to whole‐brain post‐mortem MRI data
Source: Neuroimage. 2023 Jan;265:119792. doi: 10.1016/j.neuroimage.2022.119792 (PMC10933796; doi:10.1016/j.neuroimage.2022.119792)
Supplement: Supplementary file 3 [file mmc3.docx]

**Supplementary Material 3 –**

**Stage-3 simulation experiment with MIND and NMI cost functions**

Figure C.1 shows that for oblique quadratic simulated slices with no Gaussian noise, MIND already outperforms NMI with respect to the final registration error (substage 4), which becomes even more obvious when Gaussian noise is added, mimicking the conditions of registering a slice of a different modality (e.g., a photograph). Based on the result of this experiment, NMI was dropped from the Stage-3 routine despite faster computations versus MIND.

**Figure C.1.** Comparison of the MIND and the normalised mutual information (NMI) image dissimilarity metric for Stage-3 registration of simulated slices (oblique quadratic series, using 16 control points in Steps 3 and 4). The registration substages are as described in the Stage-3 algorithm: 0) perturbed initial state, 1) rigid, 2) affine, 3) in-plane deformation, 4) 3D deformation.
