# Supplementary material for: Tensor image registration library: Deformable registration of stand‐alone histology images to whole‐brain post‐mortem MRI data
Source: Neuroimage. 2023 Jan;265:119792. doi: 10.1016/j.neuroimage.2022.119792 (PMC10933796; doi:10.1016/j.neuroimage.2022.119792)
Supplement: Supplementary file 4 [file mmc4.docx]

**Supplementary Material 4 –**

**Effect of regularisation weight on Stage-1 registration results: a visual comparison**


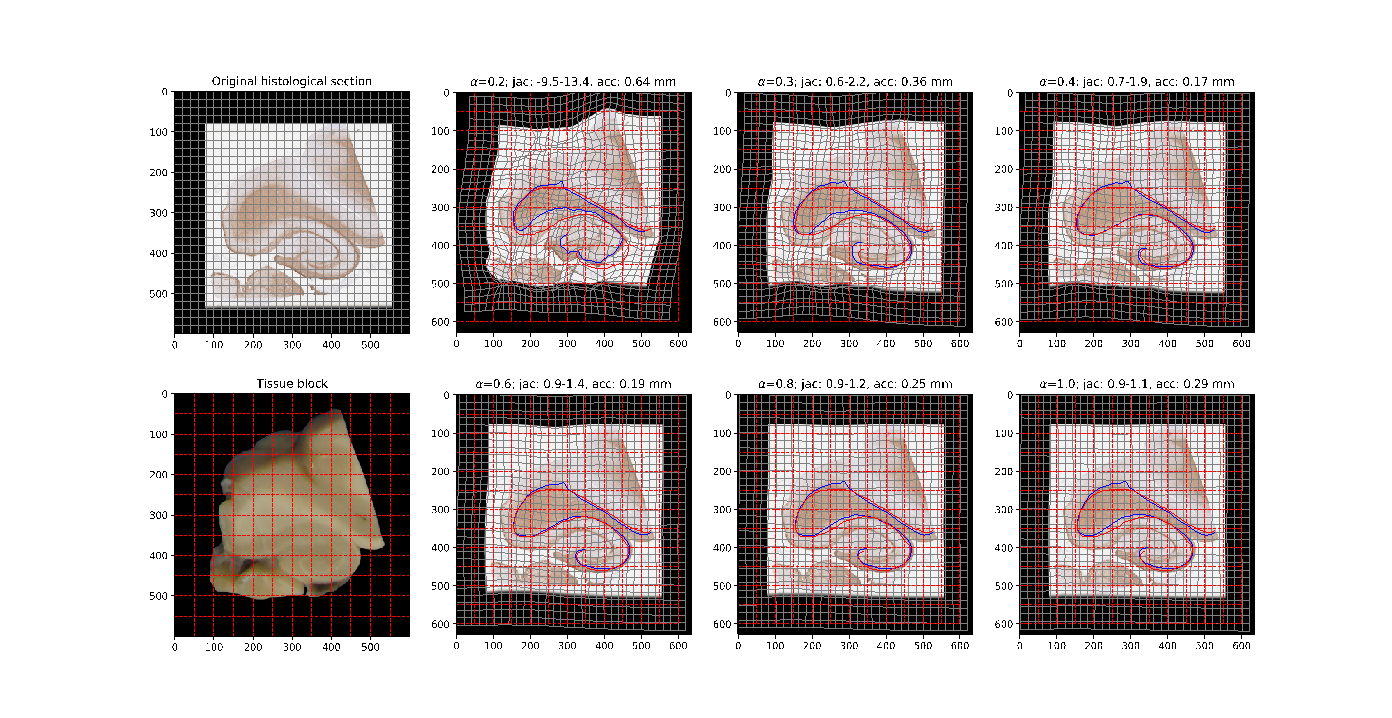


**Figure D.1. Stage-1 registration results on a hippocampal section using varying weights (0.2 < α < 1.0) for diffusion regularisation.** Images show deformed versions of the histology image on the domain of the tissue block photograph after the registration. The *blue curve* represents the transformed grey-white matter contour of the histology image, and the *red curve* is the grey-white matter boundary as observed in the tissue block photo. Their median distances are reported in millimetres above the images (*acc*). The Jacobian range (*jac*) is calculated from the total deformation of the histology image, and it indicates the magnitude of the largest local compression and largest local dilation of the image in relative units (1 = no compression).

Figure D.1 suggests an optimal range for α between 0.4 and 0.6, corresponding to slightly more conservative deformations at α = 0.6 based on the Jacobian ranges. All values of α, except for 0.2 led to diffeomorphic transformations with Jacobians > 0.
