# Supplementary material for: Tensor image registration library: Deformable registration of stand‐alone histology images to whole‐brain post‐mortem MRI data
Source: Neuroimage. 2023 Jan;265:119792. doi: 10.1016/j.neuroimage.2022.119792 (PMC10933796; doi:10.1016/j.neuroimage.2022.119792)
Supplement: Supplementary file 5 [file mmc5.docx]

**Supplementary Material 5 – Histology artefact masking**

**Defining artefact masks**

For all stages of the pipeline, image masks may be defined via a uniform interface in the stage-specific configuration files:

*# Specify or auto-generate a cost function mask for the slice image.*mask:
 *# Specify mask image file. The mask image must have the same aspect*

*# ratio as the input image. No mask: null.* file: NP082_16/slices/13B/070_crop_seg_handmask.tif
 *# Create a binary mask by segmenting the image using both a lower and an
 # upper threshold value. Values are relative to the maximum intensity.
 # Pixels with intensities lower than the thr, or higher than the uthr*

*# will be excluded from the registration. No mask: thr=0, uthr=1.* automask: {thr: 0.1, uthr: 1.0}
 *# Execute a function to generate a mask. The function must be defined in
 # the registration script under the same name as it is referenced by*

*# here. See the source code for function templates. No mask: null.* function: null

Through the “mask interface”, the user can define image masks in three different ways:

- *by loading it from an image file*: depending on the image format, mask values may be of binary, integer, or floating-point type
- *by thresholding the image at a specific lower and/or upper pixel intensity*: creates a binary mask, which excludes areas where the pixel intensity falls outside the specified relative pixel intensities
- *by executing a custom function*: users may supplement the Python script file with a custom function that computes the mask for the image, e.g., to create a dilated object mask

**Our recommendations for defining masks:**

- binary masks are usually sufficient, continuous values may add unnecessary complexity
- use masks sparingly, only to exclude artefacts that are not represented in the other image (e.g., a missing piece of tissue)
- simple geometric forms, such as a circle or rectangle are usually adequate to create exclusion zones
- do not exclude significant edges, such as object boundaries, unless they are artificial and not matched by an analogous edge in the other image

**Visualising TImage masks as overlays**

The pipeline scripts generate a separate TImage file for each input image. Each TImage file encapsulates the image data, the optimised transformations of the image, as well as the any mask that was specified for the image. We created a command-line utility to visualise TImage masks as a semi-transparent colour overlay on top of the TImage data. For example:

tirl mask.overlay path/to/registration/folder/fixed4_nonlinear.timg --threshold 0.5 --show

The above command (with a suitable masked TImage input) displays the following Matplotlib figure (red: excluded areas, blue: included areas):


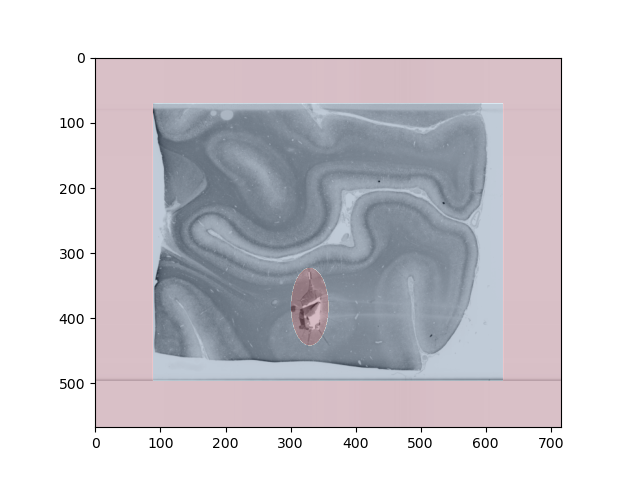


**The effect of artefact masking**

We present three discarded histology images from the dataset that will hopefully set a realistic expectation for the capabilities and limitations of artefact masking.

From left to right, the examples show the histology image with the artefact mask (1), the tissue block photograph as the registration target (2), the result of registering the histology image with (3) and without the mask (4), and the displacement field (5) to map the masked registration result to the unmasked registration result. The colours of the displacement field represent direction and the intensity of the colours is proportional to the magnitude of the local displacement. We give the largest displacement in numeric format.

**Case 1: small, circumscribed artefact 🡪 the effect of masking is minimal**

**Case 2: missing tissue at the boundary of the specimen 🡪 masking is most effective**

**Case 3: tear with dislocation, affecting the overall shape of the specimen 🡪 masking is virtually ineffective**
